# The Potential of Serum Exosomal hsa_circ_0028861 as the Novel Diagnostic Biomarker of HBV-Derived Hepatocellular Cancer

**DOI:** 10.3389/fgene.2021.703205

**Published:** 2021-07-23

**Authors:** Yuanxiao Wang, Lin Pei, Zhihong Yue, Mei Jia, Hui Wang, Lin-Lin Cao

**Affiliations:** Department of Clinical Laboratory, Peking University People’s Hospital, Beijing, China

**Keywords:** hepatocellular cancer, hepatitis B virus, exosome, circular RNA, hsa_circ_0028861

## Abstract

Hepatitis B virus (HBV)-derived hepatocellular cancer (HCC) is a serious threat to human health, especially in China. There is no highly sensitive and specific HCC biomarker at present, which makes it difficult to detect HCC at the early stage. Serum exosomal circular RNAs (circRNAs) have been reported as novel diagnostic and prognostic biomarkers of cancers. In the present study, we aimed to explore the diagnostic performance of serum exosomal circRNAs for HBV-derived HCC screening. At first, many circRNAs were found to be differentially expressed in the serum exosomes of HCC individuals by microarray analysis. The validation of dysregulated circRNAs by qRT-PCR revealed that serum exosomal hsa_circ_0028861 was decreased in HCC compared to chronic HBV and cirrhosis. Then, hsa_circ_0028861 was identified as a novel biomarker for HCC diagnosis with an area under the ROC curve (AUC) of 0.79 for discriminating HCC from chronic HBV and cirrhosis individuals. Hsa_circ_0028861 was capable of detecting small (AUC = 0.81), early-stage (AUC = 0.82) and AFP-negative [AFP (−)] (AUC = 0.78) tumors as well. The combination of hsa_circ_0028861 and AFP exhibited better diagnostic ability (AUC = 0.86 for discriminating HCC from chronic HBV and cirrhosis). Moreover, bioinformatics prediction suggested that hsa_circ_0028861 might influence HCC progression by regulating its targeted microRNAs (miRNAs) and downstream tumor-related signaling pathways. Collectively, our study reveals a novel diagnostic tool for HBV-derived HCC.

## Introduction

Hepatocellular cancer (HCC) is a malignant tumor that seriously threatens human health. Its morbidity ranks sixth, and its mortality ranks fourth worldwide (Bray et al., 2018). It has been widely known that some factors, such as hepatitis B virus (HBV), hepatitis C virus (HCV), and alcohol, are independent etiological risk factors for HCC (Ghouri et al., 2017), but the molecular mechanism of HCC development is not fully understood yet. Due to the insidious process of HCC, patients are usually diagnosed at an advanced stage. Currently, serum alpha-fetoprotein (AFP) is the most widely used clinical marker for HCC diagnosis, but its diagnostic sensitivity and specificity are limited (Forner et al., 2012). Therefore, it is critical to explore novel diagnostic markers for HCC.

Exosomes are small extracellular vesicles that contain various substances such as proteins, lipids, and nucleic acids (Jeppesen et al., 2019). It has been documented that exosomes are involved in the biological processes and pathogenesis of a variety of diseases including cancer (Malm et al., 2016; Sato et al., 2016; Xu et al., 2018). Exosomes play a critical role in regulating cell–cell communication *via* transmitting some molecules, such as nucleic acids and proteins, and thus influence tumor cell growth, angiogenesis, metastasis, immune response, and other biological processes (Umezu et al., 2014; Becker et al., 2016; Bhagirath et al., 2018; Chen et al., 2018; Xie et al., 2019). In recent years, it has been reported that circular RNA (circRNA) can be stably present in exosomes and play a crucial role in cancer progression (Li et al., 2015; Zhang et al., 2019).

circRNA is a type of endogenous RNA with a covalent closed loop structure (Lasda and Parker, 2014). It is not easily hydrolyzed by RNase R due to the lack of 5' cap and 3' poly(A) tail and has better stability than linear RNA (Suzuki and Tsukahara, 2014). It exhibits a certain degree of conservation, and many circRNAs are only expressed in specific cell type or developmental stage (Jeck et al., 2013; Memczak et al., 2013), suggesting their critical roles in physiological or pathological processes. circRNA can act as microRNA (miRNA) “sponge,” and regulates gene expression by targeting the corresponding miRNA (Memczak et al., 2013). Many circRNAs have been shown to sponge miRNAs, such as ciRS-7 (circular RNA sponge for miR-7; Hansen et al., 2013), circMTO1 (circular RNA sponge for miRNA-9; Han et al., 2017), and circTMEM45A (circular RNA sponge for miRNA-665; Zhang et al., 2020). Numerous studies have shown that circRNAs play important roles in the initiation and progression of tumors including HCC (Meng et al., 2017). For example, exosomal circPTGR1 from highly metastatic HCC cells could promote the migration and invasion of lower-metastatic cells *via* the miR449a-MET pathway, thereby destroying the homeostasis of tumor microenvironment and facilitating HCC progression (Wang et al., 2019). In addition, circMTO1 inhibited the progression of HCC by adsorbing miRNA-9 and promoting the expression of tumor suppressor p21 (Han et al., 2017). However, the roles of circRNAs in the progression of HBV-derived HCC and their potential as diagnostic markers remain unclear.

As circRNAs can stably exist in serum exosomes of peripheral blood (Li et al., 2015), they may serve as potential disease markers that can be applied in clinical practices. In this study, we aimed to determine the clinical value of serum exosomal circRNAs in HBV-derived HCC. Serum exosomes of peripheral blood were extracted, and circRNA microarray was used to screen exosomal circRNAs that were differentially expressed in HBV-derived HCC compared with chronically infected HBV patients. We identified that serum exosomal hsa_circ_0028861 was aberrantly expressed in HCC. Then its diagnostic value and its correlation with the clinicopathologic variables of HCC patients were also evaluated. Our study suggests that serum exosomal hsa_circ_0028861 can be used as a novel biomarker for HCC diagnosis.

## Materials and Methods

### Study Population

A total of 56 HCC patients, 47 patients with liver cirrhosis, and 57 chronically infected HBV patients were recruited in Peking University People’s Hospital. Each patient with HCC or cirrhosis had a history of chronic HBV infection. Chronic HBV infection was confirmed by the previous medical history and detection of HBV immunological markers. Patients with liver cirrhosis were confirmed by imaging, and HCC patients were confirmed by histopathology. Peripheral blood samples were collected before surgery. The implementation of this study complied with the principles of the Declaration of Helsinki and was approved by the Ethics Committee of Peking University People’s Hospital. Informed consents were signed by all recruited patients. The clinicopathological characteristics were obtained from medical records and are summarized in Tables 1 and 2.

**Table 1:** The clinical variables of individuals included in this study.

Variables	HBV (*N* = 57)	CIRC (*N* = 47)	HCC (*N* = 56)	*p*
Age				
≤50 y	21	14	13	0.287
>50 y	36	33	43	
Gender				
Male	38	35	45	0.253
Female	19	12	11	
AFP				
>7 ng/ml	10	20	34	<0.0001
≤7 ng/ml	45	27	21	

**Table 2:** Correlation of clinicopathological variables with the expression of hsa_circ_0028861 in serum exosomes from HCC patients.

Variables	*N*	hsa_circ_0028861 expression		*p*
		Low	High	
Age				
≤50 y	13	6	7	1.000
>50 y	43	22	21	
Gender				
Male	45	23	22	1.000
Female	11	5	6	
AFP				
>7 ng/ml	34	16	18	0.785
≤7 ng/ml	21	11	10	
Tumor size				
>5 cm	25	14	11	0.591
≤5 cm	31	14	17	
Clinical stage				
I-II	22	10	12	0.785
III-IV	34	18	16	
Differentiation				
High-moderate	29	17	12	1.000
Low	7	4	3	
Vascular invasion				
Yes	29	16	13	0.593
No	27	12	15	
Encapsulation invasion				
Yes	15	8	7	1.000
No	41	20	21	
Tumor number				
=1	24	13	11	0.787
>1	32	15	17	
Lymph node metastasis				
Yes	18	11	7	0.391
No	38	17	21	
Distant metastasis				
Yes	10	6	4	0.729
No	46	22	24	

### Exosome Isolation and Identification

Serum exosomes were isolated using an ExoQuick Ultra EV Isolation Kit for Serum and Plasma (System Biosciences, Cat: EQULTRA-20A-1) according to the manufacturer’s User Manual. Then, the morphology of the exosomes was observed by transmission electron microscope (TEM), and the size distribution was determined by nanoparticle tracking analysis (NTA) as described before (Zhang et al., 2019). Specific protein markers of exosomes were detected by western blot.

### Western Blot

Exosomes were lysed with RIPA Lysis Buffer I (Sangon Biotech, Cat: C500005) to obtain the total protein. Then, the extracted proteins were separated by sodium dodecyl sulfate polyacrylamide gel electrophoresis (SDS-PAGE) and transferred onto a nitrocellulose membrane. The primary antibodies used here included: anti-CD9 (Abclonal, Cat: A1703), anti-CD63 (Abclonal, Cat: A5271), anti-TSG101 (Abclonal, Cat: A1692), and anti-HSPA5 (Abclonal, Cat:A11366).

### RNA Extraction

TRIzol Reagent (Invitrogen, Cat: 15596026) was used to extract total RNA in exosomes according to the manufacturer’s instructions. The concentration and OD260/OD280 ratio of the extracted RNA were measured using a NanoDrop spectrophotometer (ThermoFisher Scientific). The OD260/OD280 ratios should be in the range of 1.8–2.0 to ensure the superior purity of the RNA samples.

### circRNA Microarray Analysis

Arraystar Human circRNA Array v2 (8x15K, Arraystar) was used to screen aberrantly expressed circRNAs in HCC. Sample preparation and microarray hybridization were performed according to the manufacturer’s standard protocols. Briefly, total RNAs were digested with RNase R (Epicenter) to remove linear RNAs, and the enriched circRNAs were amplified and transcribed into fluorescent compliment RNAs (cRNAs) with random primers using a Super RNA Labeling Kit (Arraystar). Then, the labeled cRNAs were hybridized onto the microarray. After washing the slides, the arrays were scanned by the Agilent Scanner G2505C, and acquired array images were analyzed by Feature Extraction software version 11.0.1.1 (Agilent). Data processing was performed using the R software limma package. Differentially expressed circRNAs between two groups were identified *via* Volcano Plot filtering. Hierarchical Clustering was performed to show the different circRNA expression pattern among samples.

### Quantitative Real-Time PCR

Equal amounts of RNA were reverse transcribed with random primers using Quantscript RT Kit (Tiangen, Cat: KR103). Quantitative real‑time PCR (qRT-PCR) of synthesized complementary DNA (cDNA) was performed on an Applied Biosystems 7500 System (ThermoFisher Scientific) using TB Green® Premix Ex Taq™ II (Takara, Cat: RR820). The primers used here were as follows: hsa_circ_0023988 (F: TGCTGACGTTGCATGTTTCAG; R: GGTTCTCCTGCTTGGAACCT); hsa_circ_0028861 (F: CTAAAATCTCCAGGGGCACCA; R: GACTTCACATGGGGCAATGG); hsa_circ_0127767 (F: ACGCAAAACGAAGACAGATCA; R: CCTGTCTGTGTCCTCCAATCC); and GAPDH (F: AAGGTCGGAGTCAACGGATTTG; R: CCTGGAAGATGGTGATGGGATT).

### Prediction of Target miRNAs for circRNA and Biological Pathway Enrichment Analysis

To further study the molecular mechanism of circRNA in HCC, the circRNA/miRNA interaction was predicted using Arraystar’s miRNA target prediction software, and the differentially regulated circRNAs were annotated in detail with the information of their targeted miRNAs. TargetScan Release 7.2^1^ (Agarwal et al., 2015), miRWalk 2.0^2^ (Sticht et al., 2018) and miRDB^3^ (Chen and Wang, 2020) were used to predict the miRNA-mRNA interaction. FunRich Version 3.1.3^4^ (Fonseka et al., 2020), which is a stand-alone gene function enrichment analysis software, was used to investigate the potential biological pathways of targeted mRNAs.

### Statistical Analysis

Statistical analyses were performed with GraphPad Prime 5.01 (GraphPad Software) or SPSS 20.0 software (IBM). All data were displayed as mean ± SD. Student’s *t* test was used to examine the differences between groups for data that followed the Gaussian distribution; otherwise Mann–Whitney *U* test was used. Receiver operating characteristic (ROC) curves were constructed and the area under the ROC curves (AUCs) were calculated to evaluate the diagnostic performance of serum exosomal hsa_circ_0028861, and the optimal sensitivity and specificity were determined at the maximum Youden’s Index. The value of *p* < 0.05 was considered as statistically significant.

## Results

### Characterization of Serum Exosomes From Chronic HBV, Cirrhosis, and HCC Individuals

**Figure 1:**
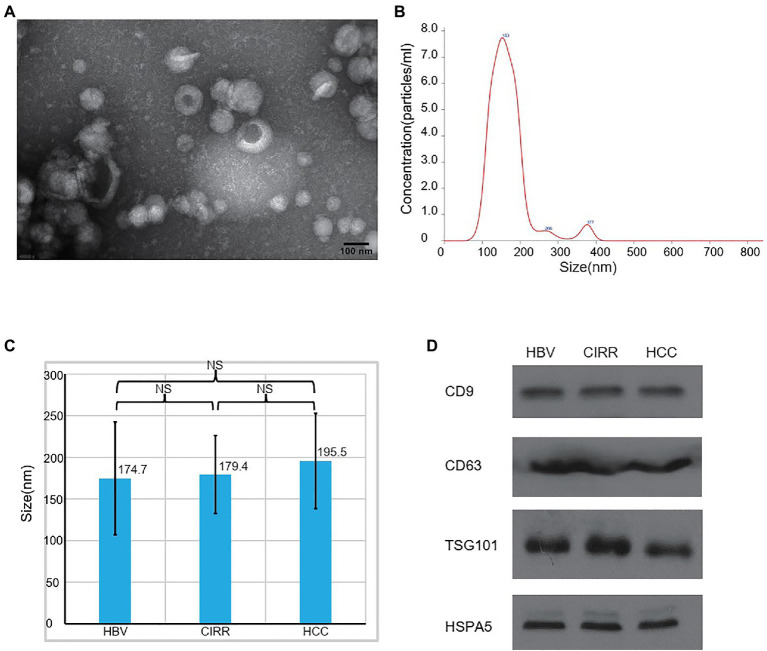
Characterization of serum exosomes of chronic HBV, cirrhosis, and HCC individuals. **(A)** A representative TEM image of exosomes; **(B)** the size range of the exosomes revealed by NAT analysis; **(C)** the histogram showing the difference of the size range between groups. **(D)** WB analysis of exosomal markers including CD9, CD63, TSG101, and HSPA5. HBV, hepatitis B virus; CIRR, cirrhosis; HCC, hepatocellular cancer; and NS, not significant.

Exosomes isolated from serum of chronic HBV, cirrhosis, and HCC individuals were verified by TEM, NTA, and western blot. As shown in Figure 1A, these particles showed the typical cup-shaped morphology by TEM. NTA analysis revealed that the diameters of most particles were in the range of 50–250 nm (Figure 1B), and there was no significant difference in the particle size of the three groups (Figure 1C). In addition, the presence of the exosome-associated markers, including CD9, CD63, TSG101, and HSPA5, was confirmed by western blot (Figure 1D). Together, these data suggested that the particles we isolated from serum were identified as exosomes.

### circRNA Microarray Analysis Identified Significantly Dysregulated Serum Exosomal circRNAs in HCC

**Figure 2:**
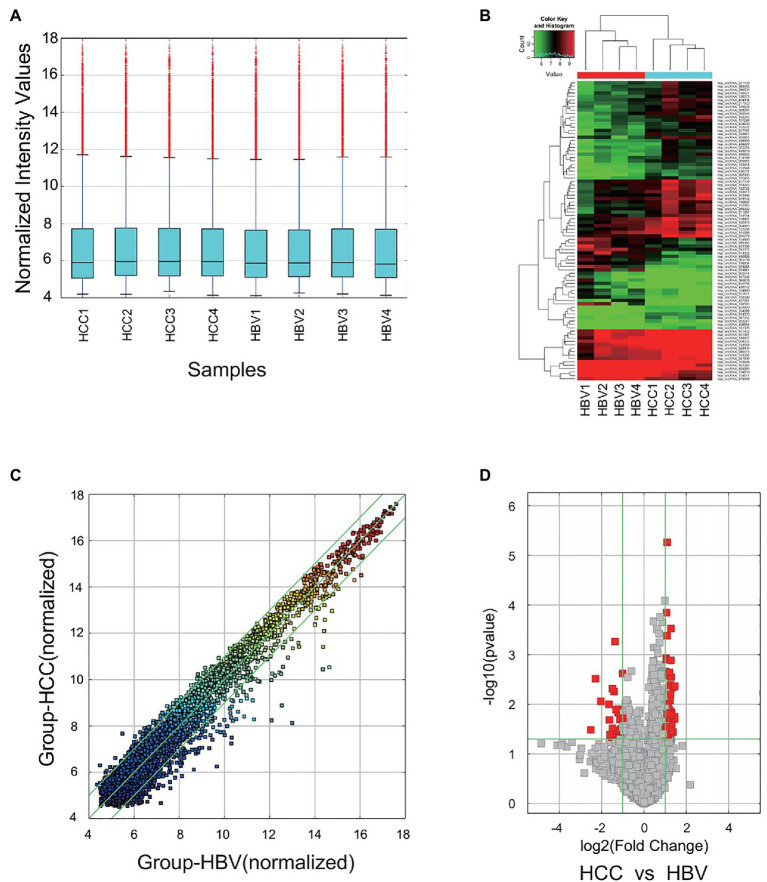
Microarray profiling identified dysregulated circRNAs between chronic HBV and HCC patients. **(A)** Boxplot of microarray data upon normalization. **(B)** Differentially expressed circRNAs between four chronic HBV and four HCC patients. Each column represents a sample, and each row represents a circRNA. “Red” indicates increased expression, and “green” indicates decreased expression. **(C)** The scatter plot assessing the circRNA expression variation between chronic HBV and HCC patients. The circRNAs above the top green line and below the bottom green line indicated fold change >2.0. **(D)** The volcano plot depicts the difference of circRNAs between chronic HBV and HCC patients. HBV, hepatitis B virus and HCC, hepatocellular cancer. Red points refer to significant dysregulation according to fold change >2.0 and *p* < 0.05.

To explore the differences in the expression profile of circRNAs in serum exosomes of patients with chronic HBV, cirrhosis, and HCC, we performed circRNA microarray analysis (Figure 2A). A total of 88 differentially expressed circRNAs were identified including 62 upregulated and 26 downregulated in HCC group. The dysregulated circRNAs and the corresponding fold change (FC) and the value of *p* are listed in Supplementary Table 1 (FC > 2.0, *p* < 0.05). The result of hierarchical clustering showed the distinguishable circRNA expression profiling between HCC and chronic HBV group (Figure 2B). In addition, a scatter plot and a volcano plot were used for visualizing the dysregulated circRNAs (Figures 2C,D).

### Validation of Dysregulated circRNAs by qRT-PCR and the Diagnostic Performance of hsa_circ_0028861

**Figure 3:**
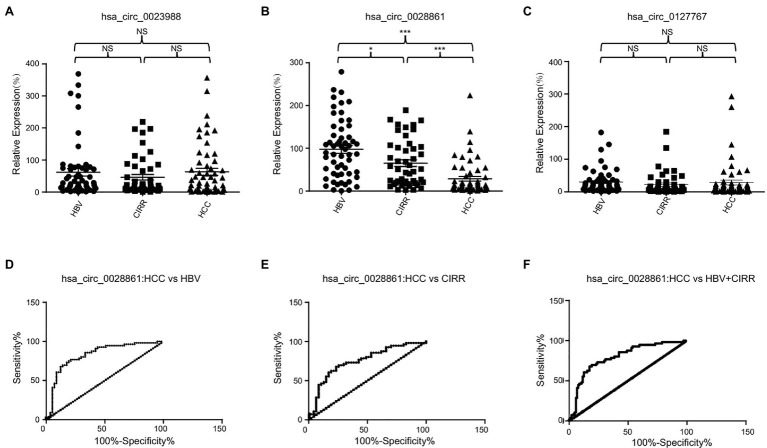
The diagnostic performance of hsa_circ_0028861. **(A–C)** The expression profile of serum exosomal hsa_circ_0023988 **(A)**, hsa_circ_0028861 **(B)** and hsa_circ_0127767 **(C)** in chronic HBV, cirrhosis, and HCC patients. **(D–F)** The ROC curves of hsa_circ_0028861 for discriminating HCC from chronic HBV **(D)**, cirrhosis **(E)** or the combination of chronic HBV and cirrhosis patients **(F)**, respectively. HBV, hepatitis B virus; CIRR, cirrhosis; HCC, hepatocellular cancer; ROC, receiver operating characteristic; and NS, not significant. ^*^p < 0.05; ^***^p < 0.001.

Subsequently, we selected one upregulated (hsa_circ_0127767) and two downregulated circRNAs (hsa_circ_0023988 and hsa_circ_0028861) that were the most differentially expressed in HCC for validation using qRT-PCR. Consistently, the expression of hsa_circ_0028861 was significantly decreased in the serum exosomes of HCC patients compared to the chronic HBV and cirrhosis individuals (Figure 3B). However, there was no significant difference in the expression levels of hsa_circ_0023988 and hsa_circ_0127767 among the three groups (Figures 3A,C). The correlations between hsa_circ_0028861 and the clinicopathological characteristics of HCC individuals are listed in Table 2.

Then we evaluated the potential diagnostic value of hsa_circ_0028861 in HCC using ROC curves. As shown in Figure 3D and Table 3, hsa_circ_0028861 exhibited an AUC of 0.83 in discriminating HCC patients from chronic HBV patients, and the corresponding sensitivity and specificity were 76.79 and 78.95%, respectively. In addition, hsa_circ_0028861 exhibited an AUC of 0.75 in discriminating HCC patients from cirrhosis patients, and the corresponding sensitivity and specificity were 67.86 and 76.60%, respectively (Figure 3E and Table 3). Moreover, hsa_circ_0028861 also exhibited good diagnostic performance with an AUC of 0.79 in discriminating HCC patients from the combination of chronic HBV and cirrhosis patients, and the corresponding sensitivity and specificity was 67.86 and 82.69%, respectively (Figure 3F and Table 3). These results suggested that hsa_circ_0028861 was dysregulated in HCC and showed good diagnostic performance for the detection of HCC.

**Table 3:** The diagnostic performance of has_circ_0028861 for HCC detection.

Groups	AUC (95% CI)	Sensitivity (%)	Specificity (%)	*p*
HCC vs. HBV	0.83 (0.75–0.91)	76.79	78.95	<0.0001
HCC vs. CIRR	0.75 (0.66–0.85)	67.86	76.60	<0.0001
HCC vs. HBV + CIRR	0.79 (0.72–0.87)	67.86	82.69	<0.0001

### The Diagnostic Performance of hsa_circ_0028861 in Small, Early-Stage, and AFP-Negative HCC

**Figure 4:**
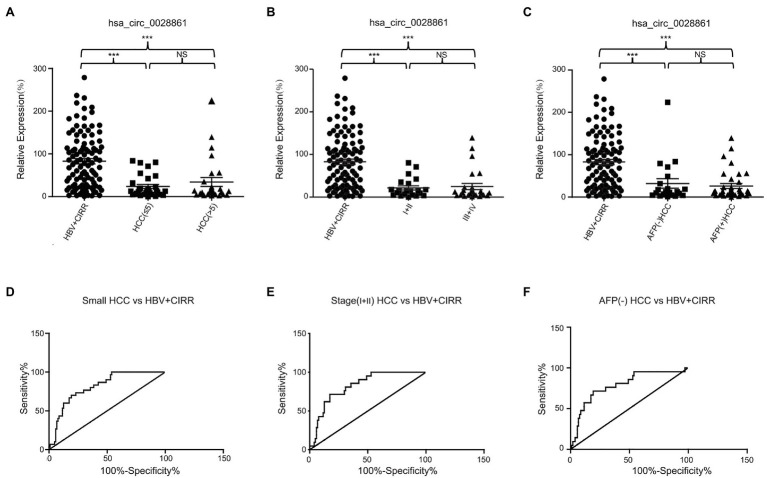
The role of hsa_circ_0028861 in the diagnosis of small, early-stage, and AFP (−) HCC patients. **(A)** The expression profile of serum exosomal hsa_circ_0028861 in small (size ≤ 5) and large (size > 5) tumors compared with chronic HBV and cirrhosis individuals. **(B)** The expression profile of serum exosomal hsa_circ_0028861 in early-stage (stage I-II) and late-stage (size III-IV) tumors compared with chronic HBV and cirrhosis individuals. **(C)** The expression profile of serum exosomal hsa_circ_0028861 in AFP (−) and AFP (+) tumors compared with chronic HBV and cirrhosis individuals. **(D–F)** The ROC curves of hsa_circ_0028861 for discriminating small **(D)**, early-stage **(E)**, and AFP (−; **F**) HCC patients. HBV, hepatitis B virus; CIRR, cirrhosis; HCC, hepatocellular cancer; ROC, receiver operating characteristic; and NS, not significant. ^***^p < 0.001.

As small, early-stage, and AFP-negative [AFP (−)] HCC patients are often difficult to detect, we evaluated the diagnostic value of hsa_circ_0028861 in these tumors. As shown in Figures 4A,D and Table 4, hsa_circ_0028861 exhibited an AUC of 0.81 in discriminating small HCC patients from the combination of chronic HBV and cirrhosis patients, and the corresponding sensitivity and specificity was 70.00 and 80.77%, respectively. In addition, hsa_circ_0028861 exhibited an AUC of 0.82 in discriminating early-stage (stage I-II) HCC patients from the combination of chronic HBV and cirrhosis patients, and the corresponding sensitivity and specificity was 71.43 and 82.69%, respectively (Figures 4B,E and Table 4). Moreover, hsa_circ_0028861 also exhibited good diagnostic performance with an AUC of 0.78 in discriminating AFP (−) HCC patients from the combination of chronic HBV and cirrhosis patients, and the corresponding sensitivity and specificity was 71.43 and 80.77%, respectively (Figures 4C,F and Table 4). Collectively, these findings indicated the importance of hsa_circ_0028861 in the diagnosis of small, early-stage, and AFP (−) HCC.

**Table 4:** The diagnostic performance of has_circ_0028861 for the detection of AFP-negative, small and early-stage HCC.

Groups	AUC (95% CI)	Sensitivity (%)	Specificity (%)	*p*
AFP (−) HCC vs. HBV + CIRR	0.78 (0.67–0.90)	71.43	80.77	<0.0001
Small HCC vs. HBV + CIRR	0.81 (0.73–0.89)	70	80.77	<0.0001
Stage (I-II) HCC vs. HBV + CIRR	0.82 (0.74–0.91)	71.43	82.69	<0.0001

### The Combination of hsa_circ_0028861 and AFP in the Diagnosis of HCC

**Figure 5:**
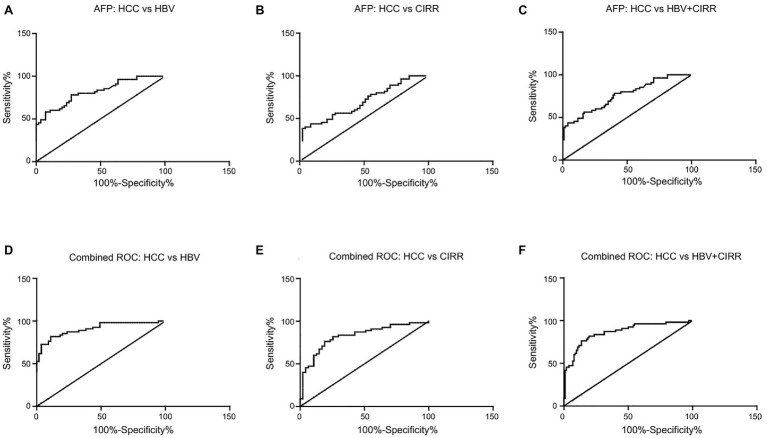
The combination of AFP and hsa_circ_0028861 for HCC diagnosis. **(A–C)** ROC curves of AFP for discriminating HCC from chronic HBV **(A)**, cirrhosis **(B)** or the combination of chronic HBV and cirrhosis patients **(C)**, respectively. **(D–F)** ROC curves of the combination of hsa_circ_0028861 and AFP for discriminating HCC from chronic HBV **(D)**, cirrhosis **(E)**, or the combination of chronic HBV and cirrhosis patients **(F)**, respectively. HBV, hepatitis B virus; CIRR, cirrhosis; and HCC, hepatocellular cancer.

As the classical HCC biomarker AFP showed a limited diagnostic efficacy, we next determined whether the combination of hsa_circ_0028861 and AFP could improve the accurate diagnosis rate of HCC. As shown in Figure 5, the combination of hsa_circ_0028861 and AFP exhibited better diagnostic performance than AFP alone in differentiating HCC patients from chronic HBV patients (AUC: 0.91 vs. 0.82; Figures 5A,D and Table 5), cirrhosis patients (AUC: 0.82 vs. 0.69; Figures 5B,E and Table 5), or the combination of chronic HBV and cirrhosis patients (AUC: 0.86 vs. 0.76; Figures 5C,F and Table 5). These results indicated that the combination of hsa_circ_0028861 and AFP might be a good diagnostic tool for HCC.

**Table 5:** The combination of has_circ_0028861 and AFP for HCC detection.

Groups	AUC (95% CI)	Sensitivity (%)	Specificity (%)	*p*
AFP				
HCC vs. HBV	0.82 (0.74–0.90)	78.18	72.73	<0.0001
HCC vs. CIRR	0.69 (0.59–0.79)	38.18	97.87	0.0008
HCC vs. HBV + CIRR	0.76 (0.68–0.84)	43.64	96.08	<0.0001
AFP + has_circ_0028861				
HCC vs. HBV	0.91 (0.85–0.96)	81.82	89.09	<0.0001
HCC vs. CIRR	0.82 (0.74–0.91)	76.36	80.85	<0.0001
HCC vs. HBV + CIRR	0.86 (0.80–0.93)	76.36	86.27	<0.0001

### Target miRNA Prediction and Biological Pathway Enrichment Analysis

**Figure 6:**
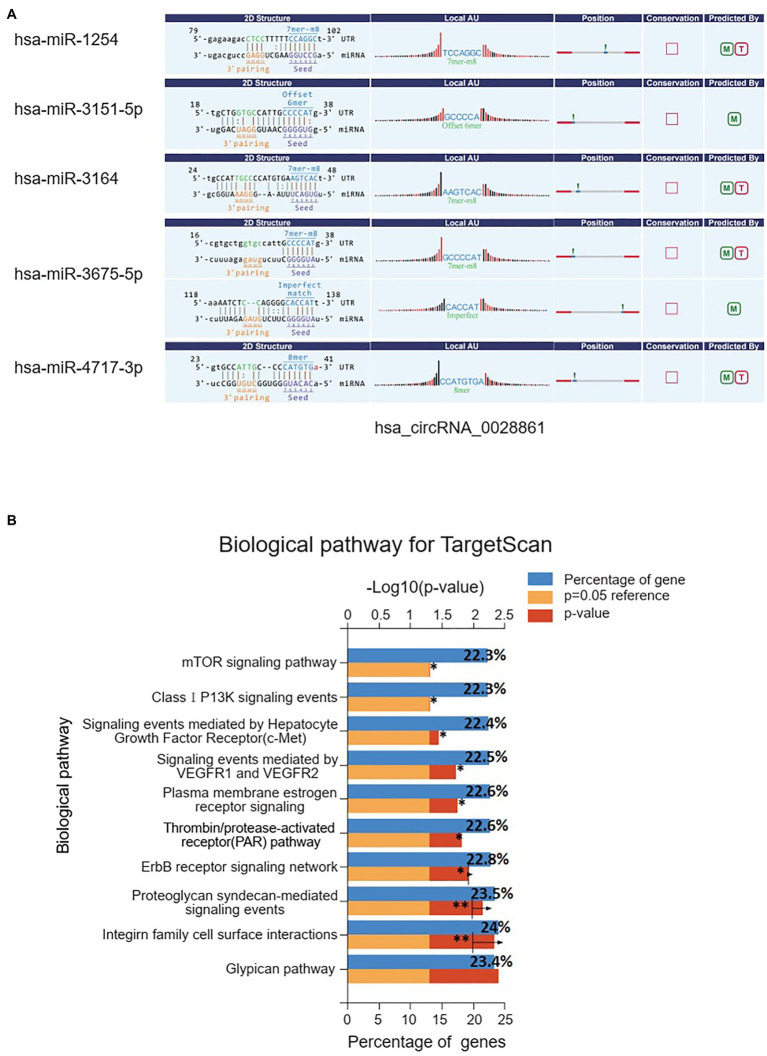
Prediction of hsa_circ_0028861 interacted miRNAs and downstream biological pathway analysis. **(A)** There were five miRNAs identified as targets of hsa_circ_0028861; **(B)** pathway enrichment analysis of the targeted mRNAs (predicted by TargetScan) of these five miRNAs suggested that hsa_circ_0028861 participated in the integrin, VEGF, PI3K/Akt and mTOR signaling pathways. ^*^p < 0.05; ^**^p < 0.01.

The prediction results from Arraystar’s miRNA target prediction software showed that hsa_circ_0028861 could interact with five miRNAs (Figure 6A). Then, we predicted the targeted mRNAs of these five miRNAs using TargetScan, miRWalk, and miRDB, respectively, and performed pathway enrichment analysis of these mRNAs using FunRich. The prediction results of the three databases were consistent, indicating that hsa_circ_0028861 played critical roles in several tumor-related signaling pathways such as integrin, vascular endothelial growth factor (VEGF), PI3K/Akt, and mTOR signaling pathways (Figure 6B and Supplementary Figure 1). Taken together, these results suggested that hsa_circ_0028861 might regulate the expression of target miRNAs, thereby modulating tumor-related signaling pathways in HCC.

## Discussion

In this study, hsa_circ_0028861 was found to be downregulated in the serum exosomes of HBV-derived HCC patients compared to chronic HBV and cirrhosis patients. Dysregulated hsa_circ_0028861 showed adequate diagnostic performance in differentiating HCC from chronic HBV and cirrhosis individuals. It also offered high-diagnostic accuracy in the detection of small, early-stage, and AFP (−) HCC. The combination of serum AFP and serum exosomal hsa_circ_0028861 had critical clinical significance in the diagnosis of HBV-derived HCC. Hsa_circ_0028861 might regulate its targeted miRNAs, leading to abnormal changes in tumor-related signal pathways and influencing HCC progression.

HCC is the most important type of primary liver cancer. It has the characteristics of high malignancy and poor prognosis (Llovet et al., 2016). In China, chronic HBV infection is the major cause of HCC. As the elimination of HBV in the human body is still difficult to achieve at present, HBV-derived HCC still poses a great health threat to the Chinese population (Zhang et al., 2017). Currently, there is no clinically effective diagnostic marker for HBV-derived HCC; and small, early-stage, and AFP (−) tumors are often difficult to detect, leading to delays in the treatment and progression of the disease. In this study, serum exosomal hsa_circ_0028861 was identified as a potential diagnostic biomarker for detecting small, early-stage, and AFP (−) HCC with high sensitivity and specificity. It will exert an important impact on the early diagnosis and prognosis improvement of HCC patients.

A growing number of studies have demonstrated that molecules in serum or plasma exosomes are of great value for tumor diagnosis including long non-coding RNAs (lncRNAs), miRNAs, and proteins (Kalluri, 2016). For example, the expression levels of long non-coding RNA UFC1 were increased in the serum exosomes of non-small cell lung cancer (NSCLC) patients compared to pneumonia patients and healthy controls, with high diagnostic sensitivity and specificity (Zang et al., 2020). In addition, serum exosomal miR-1,246 was significantly upregulated in aggressive prostate cancer and showed similar diagnostic performance as serum prostate-specific antigen (PSA), which was the classical biomarker for prostate cancer (Bhagirath et al., 2018). Moreover, protein biomarkers from serum exosomes were identified as potential diagnostic tools for primary sclerosing cholangitis and cholangiocarcinoma (Arbelaiz et al., 2017). Similarly, circRNA is also a kind of non-coding RNA that is enriched in exosomes, and the diagnostic value of exosomal circRNA in serum or plasma for cancer has been described as well. For example, serum exosomal hsa-circ-0004771 was significantly increased in colorectal cancer (CRC) patients and served as a promising diagnostic biomarker for CRC (Pan et al., 2019). In this study, our results further support the vital role of serum exosome in clinical diagnosis of cancer.

It has been reported that circRNA has a critical role in HCC diagnosis. For example, hsa_circ_0001649 was significantly reduced, while circTMEM45A, circ_104075, hsa_circ_0091579, hsa_circ_0004001, hsa_circ_0004123, hsa_circ_0075792, and hsa_circ_0001445 were significantly upregulated in HCC (Qin et al., 2016; Zhang et al., 2018a,b,c, 2020), which might serve as potential HCC biomarkers. However, the diagnostic performance of each circRNA varies greatly, and the role of circRNA in the diagnosis of HBV-derived HCC has not been depicted. Previous studies have also shown the clinical value of exosomal circRNA in serum or plasma in HCC. For example, plasma exosomal hsa_circ_0051443 appeared to be a useful marker for distinguishing HCC patients from healthy controls (Chen et al., 2020). In addition, plasma exosomal hsa_circ_0070396 was significantly increased in HCC patients, and could differentiate HCC individuals from healthy donors, patients with chronic HBV and liver cirrhosis (Lyu et al., 2021). However, neither of these two circRNAs was found significant differences between HCC and HBV group in our high-throughput microarray screening (Supplementary Table 1), which might be due to the differences in sample selection. As the aim of this study was to explore novel diagnostic markers for HBV-derived HCC, all included patients with liver cirrhosis and HCC had a history of chronic HBV infection. In this study, we found that hsa_circ_0028861 was dysregulated in HBV-derived HCC compared to chronic HBV group and might be specific for the diagnosis of HBV-derived HCC individuals.

Our further exploration revealed that hsa_circ_0028861 regulated the expression of five target miRNAs, and participated in some tumor-related signal pathways such as integrin, VEGF, PI3K/Akt, and mTOR. Previous studies have found that some signal pathways played critical roles in HCC progression. For example, dysregulated integrin expression patterns have been linked to HCC, and integrin signaling is associated with an increased risk of recurrence and gefitinib resistance (Hamidi and Ivaska, 2018). In addition, VEGF and its targeted VEGFRs have been studied in depth as the most prominent regulators of angiogenesis, which are vital for HCC growth and development (Morse et al., 2019). Moreover, alterations in the PI3K/Akt/mTOR axis are described as one of the major events in HCC, and exhibit as a crucial regulator of cancer cell proliferation and survival (Khemlina et al., 2017). In this study, we predicted that hsa_circ_0028861 could regulate the above-mentioned signal pathways through its interacted miRNAs and influence the progression of HCC, suggesting the important role of hsa_circ_0028861 in HCC.

It has been described that many circRNAs are frequently dysregulated in cancer (Meng et al., 2017). Our study is the first to demonstrate that hsa_circ_0028861 is downregulated in the serum exosomes of HBV-derived HCC patients and exhibits good diagnostic performance for discriminating HCC individuals from chronic HBV and cirrhosis individuals. Therefore, hsa_circ_0028861 can serve as a potential biomarker for HCC diagnosis. In addition, hsa_circ_0028861 is predicted as a vital regulator of HCC progression and may be a novel therapeutic target for HCC treatment. This study provides a useful tool with which to diagnose HCC and design anticancer therapies by manipulating hsa_circ_0028861.

## Data Availability Statement

The raw data supporting the conclusions of this article will be made available by the authors, without undue reservation.

## Ethics Statement

The studies involving human participants were reviewed and approved by the Ethics Committee of Peking University People’s Hospital. The patients/participants provided their written informed consent to participate in this study.

## Author Contributions

L-LC and YW conceived and designed the experiments. YW, LP, and L-LC performed the experiments. ZY, MJ, and L-LC analyzed the data. L-LC wrote the manuscript. ZY, HW, and MJ revised the manuscript. All authors contributed to the article and approved the submitted version.

### Conflict of Interest

The authors declare that the research was conducted in the absence of any commercial or financial relationships that could be construed as a potential conflict of interest.

## Footnotes

^1^http://www.targetscan.org/

^2^http://mirwalk.umm.uni-heidelberg.de/

^3^http://mirdb.org/

^4^http://www.funrich.org

## Supplementary Material

Supplementary Figure 1 **(A)** Pathway enrichment analysis of the targeted mRNAs (predicted by miRWalk) of the five miRNAs as described in Figure 6. **(B)** Pathway enrichment analysis of the targeted mRNAs (predicted by miRDB) of the five miRNAs as described in Figure 6. ^*^*p* < 0.05; ^**^*p* < 0.01; ^***^*p* < 0.001.
