# Supplementary figures and images for: The Potential of Serum Exosomal hsa_circ_0028861 as the Novel Diagnostic Biomarker of HBV-Derived Hepatocellular Cancer
Source: Front Genet. 2021 Jul 23;12:703205. doi: 10.3389/fgene.2021.703205 (PMC8345012; doi:10.3389/fgene.2021.703205)

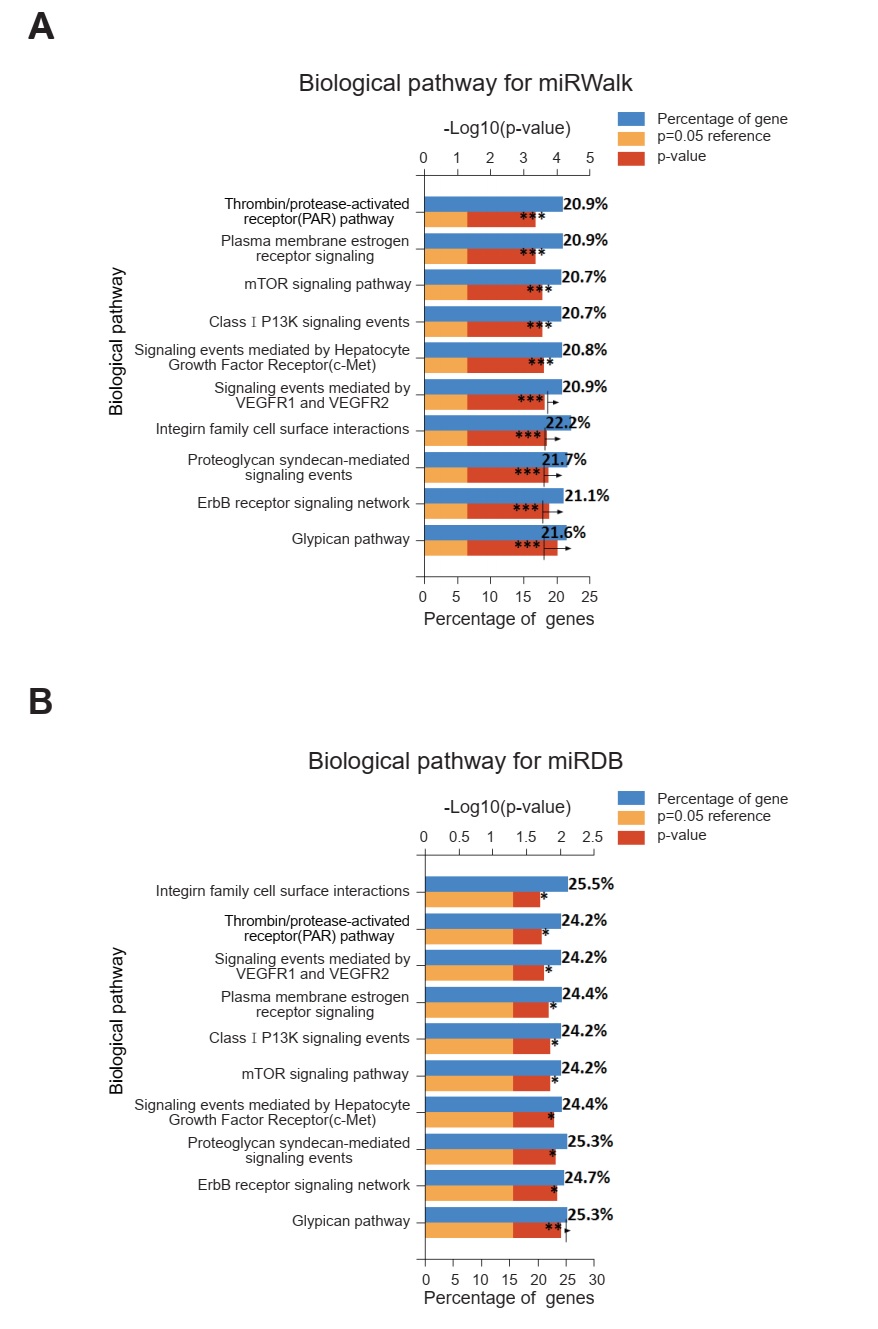

Supplement: Supplementary Figure 1 — (A) Pathway enrichment analysis of the targeted mRNAs (predicted by miRWalk) of the five miRNAs as described in Figure 6. (B) Pathway enrichment analysis of the targeted mRNAs (predicted by miRDB) of the five miRNAs as described in Figure 6. *p < 0.05; **p < 0.01; ***p < 0.001. [file Image_1.JPEG]
