# Supplementary material for: The Potential of Serum Exosomal hsa_circ_0028861 as the Novel Diagnostic Biomarker of HBV-Derived Hepatocellular Cancer
Source: Front Genet. 2021 Jul 23;12:703205. doi: 10.3389/fgene.2021.703205 (PMC8345012; doi:10.3389/fgene.2021.703205)
Supplement: Supplementary file 2 [file Table_1.DOCX]

**Supplementary Table 1. The dysregulated circRNAs in the serum exosomes of HCC patients compared to chronic HBV patients**

| circRNA | Alias | Regulation | FC (abs) | P-value |
| --- | --- | --- | --- | --- |
| hsa_circRNA_018168 | hsa_circ_0018168 | up | 2.0552657 | 0.039828 |
| hsa_circRNA_052254 | hsa_circ_0052254 | up | 2.0156928 | 0.026931 |
| hsa_circRNA_101089 | hsa_circ_0027261 | up | 2.050786 | 0.033957 |
| hsa_circRNA_102951 | hsa_circ_0058805 | up | 2.2942163 | 0.012997 |
| hsa_circRNA_402255 |  | up | 2.7293282 | 0.017764 |
| hsa_circRNA_102059 | hsa_circ_0043428 | up | 2.274258 | 0.045419 |
| hsa_circRNA_052247 | hsa_circ_0052247 | up | 2.0577592 | 0.002619 |
| hsa_circRNA_406376 |  | up | 2.0811284 | 0.000143 |
| hsa_circRNA_103548 | hsa_circ_0068514 | up | 2.0628187 | 0.001176 |
| hsa_circRNA_404954 |  | up | 2.4351448 | 0.014323 |
| hsa_circRNA_103154 | hsa_circ_0062165 | up | 2.0252995 | 0.018342 |
| hsa_circRNA_060303 | hsa_circ_0060303 | up | 2.1990568 | 0.033462 |
| hsa_circRNA_405043 |  | up | 2.1994678 | 0.023971 |
| hsa_circRNA_104389 | hsa_circ_0080436 | up | 2.3727605 | 0.004086 |
| hsa_circRNA_100373 | hsa_circ_0003039 | up | 2.0172947 | 0.002198 |
| hsa_circRNA_028935 | hsa_circ_0028935 | up | 2.3852578 | 0.019099 |
| hsa_circRNA_050545 | hsa_circ_0050545 | up | 2.1240986 | 0.042693 |
| hsa_circRNA_102296 | hsa_circ_0046854 | up | 2.1841344 | 0.034968 |
| hsa_circRNA_055953 | hsa_circ_0055953 | up | 2.7259439 | 0.019791 |
| hsa_circRNA_403966 |  | up | 2.4122844 | 0.043211 |
| hsa_circRNA_100629 | hsa_circ_0005164 | up | 2.3510083 | 0.008296 |
| hsa_circRNA_102975 | hsa_circ_0004946 | up | 2.0124327 | 0.02933 |
| hsa_circRNA_102563 | hsa_circ_0051260 | up | 2.5605993 | 0.004618 |
| hsa_circRNA_101249 | hsa_circ_0029984 | up | 2.132499 | 0.031873 |
| hsa_circRNA_103413 | hsa_circ_0004859 | up | 2.3896905 | 0.006265 |
| hsa_circRNA_403829 |  | up | 2.4206343 | 0.002827 |
| hsa_circRNA_101435 | hsa_circ_0033144 | up | 2.1153981 | 0.028133 |
| hsa_circRNA_103243 | hsa_circ_0001238 | up | 2.1162349 | 0.043977 |
| hsa_circRNA_401403 |  | up | 2.1824878 | 0.036032 |
| hsa_circRNA_104801 | hsa_circ_0087319 | up | 2.1008728 | 0.020865 |
| hsa_circRNA_100842 | hsa_circ_0022591 | up | 2.1313858 | 0.019426 |
| hsa_circRNA_404630 |  | up | 2.0088837 | 0.022514 |
| hsa_circRNA_004993 | hsa_circ_0004993 | up | 2.0786841 | 0.01018 |
| hsa_circRNA_102393 | hsa_circ_0048025 | up | 2.3562552 | 0.048123 |
| hsa_circRNA_103414 | hsa_circ_0001320 | up | 2.1243736 | 0.000415 |
| hsa_circRNA_087352 | hsa_circ_0087352 | up | 2.0696085 | 0.014048 |
| hsa_circRNA_049472 | hsa_circ_0049472 | up | 2.0687615 | 0.046915 |
| hsa_circRNA_100937 | hsa_circ_0004214 | up | 2.5811137 | 0.036247 |
| hsa_circRNA_101325 | hsa_circ_0031288 | up | 2.0665672 | 0.001248 |
| hsa_circRNA_102723 | hsa_circ_0054656 | up | 2.4848637 | 0.026039 |
| hsa_circRNA_100421 | hsa_circ_0006324 | up | 2.352505 | 0.017201 |
| hsa_circRNA_005663 | hsa_circ_0005663 | up | 2.1943047 | 0.011128 |
| hsa_circRNA_008297 | hsa_circ_0008297 | up | 2.4142651 | 0.001295 |
| hsa_circRNA_021120 | hsa_circ_0021120 | up | 2.2369625 | 0.040192 |
| hsa_circRNA_017502 | hsa_circ_0017502 | up | 2.0342762 | 0.015095 |
| hsa_circRNA_011267 | hsa_circ_0011267 | up | 2.4768939 | 0.015196 |
| hsa_circRNA_002005 | hsa_circ_0002005 | up | 2.7415021 | 0.004333 |
| hsa_circRNA_407339 |  | up | 2.1703303 | 0.039448 |
| hsa_circRNA_406654 | hsa_circ_0127767 | up | 2.1373561 | 5.4E-06 |
| hsa_circRNA_406050 |  | up | 2.1831345 | 0.019164 |
| hsa_circRNA_401091 |  | up | 2.3227574 | 0.016396 |
| hsa_circRNA_406422 |  | up | 2.1313587 | 0.032547 |
| hsa_circRNA_000302 | hsa_circ_0000302 | up | 2.0886769 | 0.041142 |
| hsa_circRNA_005279 | hsa_circ_0005279 | up | 2.3704137 | 0.034028 |
| hsa_circRNA_041939 | hsa_circ_0041939 | up | 2.0334513 | 0.034017 |
| hsa_circRNA_074535 | hsa_circ_0074535 | up | 2.4056223 | 0.000298 |
| hsa_circRNA_008323 | hsa_circ_0008323 | up | 2.0451398 | 0.040112 |
| hsa_circRNA_077101 | hsa_circ_0077101 | up | 2.0118352 | 0.032283 |
| hsa_circRNA_405010 |  | up | 2.1334769 | 0.015492 |
| hsa_circRNA_086475 | hsa_circ_0086475 | up | 2.0479235 | 0.042905 |
| hsa_circRNA_090533 | hsa_circ_0090533 | up | 2.0612855 | 0.028668 |
| hsa_circRNA_102432 | hsa_circ_0000885 | up | 2.2932912 | 0.002245 |
| hsa_circRNA_092383 | hsa_circ_0002045 | down | 2.3388525 | 0.012523 |
| hsa_circRNA_092372 | hsa_circ_0000166 | down | 2.1950816 | 0.020067 |
| hsa_circRNA_406888 |  | down | 2.3247367 | 0.014781 |
| hsa_circRNA_014352 | hsa_circ_0014352 | down | 3.0939664 | 0.020452 |
| hsa_circRNA_104860 | hsa_circ_0003965 | down | 2.3519133 | 0.034665 |
| hsa_circRNA_407340 |  | down | 2.025731 | 0.031342 |
| hsa_circRNA_102580 | hsa_circ_0051657 | down | 2.6555448 | 0.005493 |
| hsa_circRNA_002414 | hsa_circ_0002414 | down | 2.139509 | 0.031382 |
| hsa_circRNA_405756 |  | down | 2.3528325 | 0.033759 |
| hsa_circRNA_104880 | hsa_circ_0003500 | down | 2.1518078 | 0.03541 |
| hsa_circRNA_104511 | hsa_circ_0082708 | down | 2.8035788 | 0.004828 |
| hsa_circRNA_104510 | hsa_circ_0004069 | down | 2.509783 | 0.012772 |
| hsa_circRNA_101811 | hsa_circ_0039318 | down | 2.0336458 | 0.032447 |
| hsa_circRNA_405386 |  | down | 2.5632485 | 0.04891 |
| hsa_circRNA_100583 | hsa_circ_0000231 | down | 5.7365073 | 0.032551 |
| hsa_circRNA_405025 |  | down | 2.8713028 | 0.045893 |
| hsa_circRNA_028861 | hsa_circ_0028861 | down | 4.9150572 | 0.003059 |
| hsa_circRNA_016459 | hsa_circ_0016459 | down | 2.0095357 | 0.002374 |
| hsa_circRNA_407081 |  | down | 2.801976 | 0.030373 |
| hsa_circRNA_103948 | hsa_circ_0003528 | down | 2.1450696 | 0.049489 |
| hsa_circRNA_079265 | hsa_circ_0079265 | down | 4.1206216 | 0.008691 |
| hsa_circRNA_062139 | hsa_circ_0062139 | down | 3.1626245 | 0.010012 |
| hsa_circRNA_100934 | hsa_circ_0023988 | down | 2.5836883 | 0.000541 |
| hsa_circRNA_405383 |  | down | 3.0450894 | 0.040675 |
| hsa_circRNA_400772 |  | down | 2.0004569 | 0.018819 |
| hsa_circRNA_060639 | hsa_circ_0060639 | down | 3.0178486 | 0.040902 |
